# Human Periodontal Ligament Stem Cells Response to Titanium Implant Surface: Extracellular Matrix Deposition

**DOI:** 10.3390/biology10090931

**Published:** 2021-09-18

**Authors:** Guya Diletta Marconi, Luigia Fonticoli, Ylenia Della Rocca, Thangavelu Soundara Rajan, Adriano Piattelli, Oriana Trubiani, Jacopo Pizzicannella, Francesca Diomede

**Affiliations:** 1 Department of Medical, Oral and Biotechnological Sciences, University “G. d’Annunzio” Chieti-Pescara, 66100 Chieti, Italy; guya.marconi@unich.it (G.D.M.); adriano.piattelli@unich.it (A.P.); 2 Department of Innovative Technologies in Medicine & Dentistry, University “G. d’Annunzio” Chieti-Pescara, 66100 Chieti, Italy; luigia.fonticoli@unich.it (L.F.); ylenia.dellarocca@unich.it (Y.D.R.); oriana.trubiani@unich.it (O.T.); francesca.diomede@unich.it (F.D.); 3 Department of Biotechnology, Karpagam Academy of Higher Education, Coimbatore 641021, India; drsoundararajan.t@kahedu.edu.in; 4 “Ss. Annunziata” Hospital, ASL 02 Lanciano-Vasto-Chieti, 66100 Chieti, Italy

**Keywords:** osseointegration, osteogenesis, adhesion, gene expression, extracellular matrix

## Abstract

**Simple Summary:**

The extracellular matrix (ECM) is a fundamental component of tissues and organs, providing the structural and non-structural support that leads to the bone osseointegration. Understanding the mechanisms of ECM components modulation is essential for developing novel strategies for tissue engineering and regenerative medicine and in particular for the dental implant surface design. The release of ECM components by cells is the first step that leads to the early stage of bone formation. The present work is focused on the modulation of ECM components expression by human periodontal ligament stem cells (hPDLSCs) cultured on two different dental implant surfaces, sandblasted (CTRL) and dual acid-etched (TEST). The effects were evaluated by means of the morphological observations and protein and gene expression. The results demonstrated that the rough surface of titanium dental implant can enhance the expression of ECM molecules and osteogenic markers in hPDLSCs. The surface topography is of vital importance as it regulates cell response. It can be considered one of the main factors that influence the success of a dental implant. The influence of surface topography on osseointegration could lead to short healing times and a better quality of oral rehabilitation and patient life.

**Abstract:**

The major challenge for dentistry is to provide the patient an oral rehabilitation to maintain healthy bone conditions in order to reduce the time for loading protocols. Advancement in implant surface design is necessary to favour and promote the osseointegration process. The surface features of titanium dental implant can promote a relevant influence on the morphology and differentiation ability of mesenchymal stem cells, induction of the osteoblastic genes expression and the release of extracellular matrix (ECM) components. The present study aimed at evaluating the in vitro effects of two different dental implants with titanium surfaces, TEST and CTRL, to culture the human periodontal ligament stem cells (hPDLSCs). Expression of ECM components such as Vimentin, Fibronectin, N-cadherin, Laminin, Focal Adhesion Kinase (FAK) and Integrin beta-1 (ITGB1), and the osteogenic related markers, as runt related transcription factor 2 (RUNX2) and alkaline phosphatase (ALP), were investigated. Human PDLSCs cultured on the TEST implant surface demonstrated a better cell adhesion capability as observed by Scanning Electron Microscopy (SEM) and immunofluorescence analysis. Moreover, immunofluorescence and Western blot experiments showed an over expression of Fibronectin, Laminin, N-cadherin and RUNX2 in hPDLSCs seeded on TEST implant surface. The gene expression study by RT-PCR validated the results obtained in protein assays and exhibited the expression of RUNX2, ALP, Vimentin (VIM), Fibronectin (FN1), N-cadherin (CDH2), Laminin (LAMB1), FAK and ITGB1 in hPDLSCs seeded on TEST surface compared to the CTRL dental implant surface. Understanding the mechanisms of ECM components release and its regulation are essential for developing novel strategies in tissue engineering and regenerative medicine. Our results demonstrated that the impact of treated surfaces of titanium dental implants might increase and accelerate the ECM apposition and provide the starting point to initiate the osseointegration process.

## 1. Introduction

Titanium represents the gold standard for the production of endosseous dental implants due to its characteristics that make it biocompatible, resistant to corrosion and possess excellent mechanical and chemical properties. Titanium surface topography can modify the cell attachment, spreading, proliferation, orientation and protein level expression. Several studies have shown that titanium alloy implants could remain embedded permanently in the bone due to its capacity to favour osseointegration process [1]. Osseointegration or osteointegration is a direct bone–metal interface without the interposition of non-bone tissue. When there is no movement between the implant and the bone with which it has a direct contact, the implant can be considered osseointegrated. Osseointegration is the main goal to reach after implant rehabilitation, the most important features allowing the osseointegration are primary stability, the type of native bone and the surgical techniques other than the surface morphology of the used dental implant [2,3,4]. In particular, the osseointegration process after dental implant insertion is strictly related to the immuno-inflammatory reactions. The proinflammatory wound-healing phase precedes a regenerative phase in which down-regulation of inflammation and up-regulation of osteogenesis related genes occur during the early osseointegration process, in fact multinucleated giant cells appear to be an integral part of the normal osseointegration event [5,6].

Based on this, osseointegration commonly describes a clinical state that guarantees the long-term stability of prosthesis. A fundamental role in the osseointegration process is played by the formation of new blood vessels, which at the interface of the dental implants seems to be enhanced by the mesenchymal stem cells (MSCs) of the oral cavity and in particular by the human periodontal ligament stem cells (hPDLSCs) [7,8]. Oral MSCs are adult stromal cells, which are distinguished by their ability to self-renew and to give rise to different cell lines (bone, fat, chondrocytes, muscles, neurons, islet cells, liver cells) [9]. In the body, adult stem cells are responsible for regular tissue maintenance as well as regenerating new tissue parts in response to injury or disease and their business can be affected in many different ways [10,11]. Oral tissues such as dental pulp and periodontal ligament can be used as alternative sources of MSCs compared to the well-known adult tissues (bone marrow, adipose tissue, etc.) [12]. Among oral stem cells, hPDLSCs are characterized by multipotency and highly proliferative potential [13]. hPDLSCs have shown a potential angiogenic role dependent on the vascular endothelial growth factor (VEGF), which plays a key role in the implant osseointegration [14]. It is well known that a good osseointegration needs to be supported by the formation of new bone tissue, which is allowed by a good supply of nourishment and oxygen through the vessels [15]. For implant osseointegration, not only the cascade of molecular and cellular events followed by vascularization and bone remodeling are important but also the microstructure of the implant itself since it appears to influence the bone rearrangement at the bone-implant surface [16,17].

Cell adhesion is a fundamental biological process for defining cell and tissue morphogenesis. This process is regulated by cell adhesion molecules, which are transmembrane receptors linked to the cytoskeleton that allow the assembly of cells in three-dimensional tissues and their interaction with the surrounding environment [18,19]. Several studies have shown how the metabolism of osteoblasts is regulated by signalling pathways responsible for the cytoskeletal rearrangement [20]. The establishment of a correct cellular contact with the surfaces of the biomaterial and the subsequent adhesion/diffusion are the first phase of the cell-material interactions that deeply influence the successful integration of the dental implant into the host tissue [21]. The extracellular matrix (ECM) proteins are involved in the cell–material interaction processes of the implant. In particular, fibronectin glycoprotein interacts with integrins on the cell surface and mediates mechanical anchoring and the formation of focal cell–cell and cell–material adhesion contacts [22]. Thus, cell adhesion molecules support osteogenic cellular responses and the consequent healing of the dental implant.

In the current work, the expression levels of representative markers of ECM, fibronectin and laminin, vimentin, N-cadherin, a key factor in cell–cell interaction, Integrin beta-1 (ITGB1) and Focal Adhesion Kinase (FAK) have been evaluated in an vitro model of hPDLSCs seeded on two different titanium implant surfaces, CTRL and TEST. The analysis of these vital factors may offer an important platform for evaluating the biological outcomes of surface characteristics and the basis for optimizing dental implant surfaces.

The aim of the present study was to analyse the gene and protein expression levels of typical extracellular and cell–cell interaction markers and how the different expression levels may influence the performance of the titanium implants and how it could influence the osteogenic and osseointegration events.

## 2. Materials and Methods

### 2.1. Ethic Statement

The protocol and informed consent from human periodontal ligament biopsies were permitted by Medical Ethics Committee at the Medical School, “G. d’Annunzio” University, Chieti, Italy (n° 266/17.04.14). The formal consent form was signed by all subjects before the collection of the samples. The Department of Medical, Oral and Biotechnological Sciences and the Laboratory of Stem Cells and Regenerative Medicine are certified according to the quality standard ISO 9001:2008 (certificate n° 32031/15/S).

### 2.2. Cell Culture

Five human periodontal ligament biopsies were scraped from human premolar teeth of patients (aged between 18 and 25 years) in general good health conditions. The tissue was taken by scaling the roots utilizing Gracey’s curettes [23]. The samples were washed five times with phosphate buffered saline (PBS, Lonza, Basel, Switzerland) and cultured utilizing TheraPEAK™MSCGM-CD™ BulletKit serum free, chemically defined (MSCGM-CD) medium (Lonza) for the growth of human MSCs. The medium was changed twice a week and cells migrated from the tissue explants after reaching about 80% of confluence were trypsinized (Lonza) and were sub-cultured till passage 2 (P2).

### 2.3. hPDLSCs Characterization

To characterize the hPDLSCs cell population cytofluorimetric detection and osteogenic differentiation procedure were performed as previously reported [24]. The expression of CD14, CD29, CD34, CD73, CD90 and CD105 was evaluated by means FACStarPLUS flow cytometry system and the FlowJo™ software (TreeStar, Ashland, OR, USA). Then the capacity of hPDLSCs to undergo towards the osteogenic commitment was evaluated maintaining the cell culture under osteogenic conditions with the MSCBM osteogenic kit (Lonza) [25]. The medium was refreshed every three days. After 3 weeks of differentiation cells were processed and the specific staining was performed using the Alizarin red S solution. The light microscopy Leica DMIL system (Leica Microsystem, Milan, Italy) was used to capture the microphotographs. To validate the differentiation qualitative data, the expressions of osteogenic specific markers (RUNX2 and ALP) were performed by means real time polymerase chain reaction (RT-PCR) and the T-test statistical analyses was used and the data were considered significant when *p* < 0.05.

### 2.4. Dental Implants

In the present paper, two different titanium disks implant surfaces, provided by Implacil De Bortoli (São Paulo, Brazil), were used: Control surface (CTRL) and Test surface (TEST). The disks were manufactured with commercially pure titanium (ASTM F67) [26]. The surface of CTRL disks was obtained by sandblasting with a mix of titanium oxide power and then cleaned with purified water, enzymatic detergent, acetone and alcohol, whereas the TEST surface, after the same sandblasting process, was cleaned with purified water, enzymatic detergent, acetone, alcohol and then a double acid attack with acetylic acid.

### 2.5. Scanning Electron Microscopy (SEM) Analysis

Human PDLSCs were seeded on CTRL and TEST surfaces for 21 days. Then, the cells were fixed for 4 h at 4 °C in 4% Glutaraldehyde in 0.05 M phosphate buffer (pH 7.4), dehydrated in increasing ethanol concentrations. Then, samples were mounted on aluminium stubs and gold-coated in Emitech K550 sputter-coater (Emitech Ltd., Ashford, UK) before imaging through SEM (EVO 50, Zeiss, Jena, Germany) [25].

### 2.6. Confocal Laser Scanning Microscopy (CLSM) Analysis

hPDLSCs placed on CTRL and TEST samples were fixed for 10 min at room temperature (RT) with 4% paraformaldehyde in 0.1 M PBS, pH 7.4. After PBS wash, cultures were made for immunofluorescence labelling. Then, cells seeded on granules were permeabilized with 0.5% Triton X-100 in PBS, followed by blocking with 5% skimmed milk in PBS. Primary monoclonal antibodies to anti-human Fibronectin (Santa Cruz Biotechnology, Santa Cruz, CA, USA), Laminin (Santa Cruz Biotechnology), N-Cadherin (Santa Cruz Biotechnology) and RUNX2 (Santa Cruz Biotechnology) were utilized, followed by Alexa Fluor 488 green fluorescence conjugated goat anti-mouse as secondary antibodies (Molecular Probes, Invitrogen, Eugene, OR, USA). Then, the specimens were incubated with Alexa Fluor 594 phalloidin red fluorescence conjugate (Molecular Probes) to stain actin cytoskeleton. Nuclei were dyed with TOPRO (Molecular Probes). Specimens were positioned facing down on glass slides and mounted with Prolong antifade (Molecular Probes) [27]. The stained samples were evaluated using a Zeiss LSM800 META confocal system, connected to an inverted Zeiss Axiovert 200 microscope equipped with a Plan Neofluar oil-immersion objective (40×/1.3 NA). The images were taken using an argon laser beam with excitation lines at 488 nm.

### 2.7. Gene Expression

RUNX2, ALP, VIM, FN1, CDH2, LAMB1, FAK and ITGB1 mRNA expression were analysed by real-time PCR. Total RNA was isolated through the PureLink RNA Mini Kit, Ambion by Like technologies Cat. no. 12183018A according to the manufacturer’s instructions [28]. The High Capacity cDNA Reverse Transcription Kit (Applied Biosystems, Foster City, CA, USA), Part. no. 4368814 was utilized to produce cDNA. The M-MLV Reverse Transcriptase reagents (Applied Biosystems) were utilized to synthesize cDNA. Real-Time PCR was executed with the Mastercycler ep realplex real-time PCR system (Eppendorf, Hamburg, Germany). The expression levels in hPDLSCs cells cultured on CTRL were compared with the expression level in hPDLSCs on TEST titanium disk. Commercially available TaqMan Gene Expression Assays (RUNX2 Hs00231692_m1, VIMHs.PT.58.38906895, FN1 Hs.PT.58.40005963, CDH2 Hs.PT.58.26024443, LAMB1 Hs.PT.583739165, PTK2 (FAK) Hs.PT.58.524947 and ITGB1 Hs.PT.58.39883300) and the TaqMan Universal PCR Master Mix (Applied Biosystems, Foster City, CA, USA) were utilized according to standard protocols. Beta-2 microglobulin (B2M Hs99999907_m1) (Applied Biosystems) was utilized for template normalization. RT-PCR was executed in three independent experiments; duplicate determinations were performed for each specimen.

### 2.8. Protein Expression

Thirty micrograms of proteins obtained from all samples were processed as previously described [29]. Blotted membranes were incubated with the following primary antibodies: rabbit anti-RUNX2 (1:750, rabbit; Sigma-Aldrich, Milan, Italy), anti-Vimentin (1:750, rabbit; Sigma-Aldrich), anti-Laminin (1:750, rabbit; Sigma-Aldrich), anti-N-cadherin (1:750, rabbit; Sigma-Aldrich) and anti-beta-actin (1:750, mouse; Santa Cruz Biotechnology, Santa Cruz, CA, USA). After five washes in PBS containing 0.1% Tween-20, membranes were incubated for 1 h at RT with peroxidase-conjugated anti rabbit and anti-mouse secondary antibodies (1:2000; ThermoFisher Scientific, Milan, Italy). Protein expression was analysed by the enhanced chemiluminescence detection method (ECL) (Amersham Pharmacia Biotech, Milan, Italy) with photo documenter Alliance 2.7 (Uvitec, Cambridge, UK). Signals were evaluated by ECL enhancing and assessed through an UVIband-1D gel analysis system (Uvitec) [30].

### 2.9. Statistical Analysis

Data were expressed as mean and standard deviation of the recorded values. The differences among the levels of the factors under investigation were analysed by three distinct two-way-ANOVA tests, one for each experiment. Tukey tests were applied for pairwise comparisons. A value of *p* < 0.05 was considered statistically significant in all tests. For gene expression analysis, the comparative 2^−ΔΔCt^ method was used to quantify the relative abundance of mRNA and to determine the relative changes in individual gene expression (relative quantification).

## 3. Results

### 3.1. hPDLSCs Characterization

**Figure 1:**
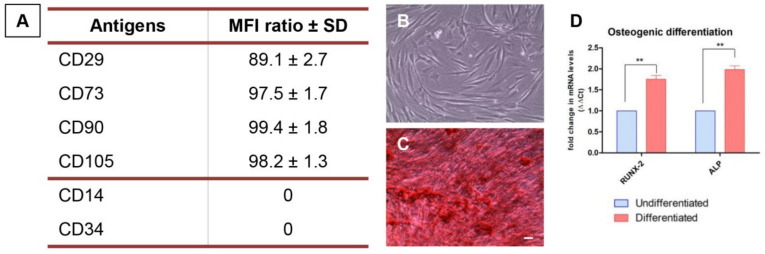
hPDLSCs characterization. (**A**) Flow cytometry results of the expression of surface markers. (**B**) Representative light microscopy image of plastic-adherent hPDLSCs cultured in standard conditions. (**C**) Cells were positive for Alizarin red S staining after 3 weeks of differentiation. (**D**) RT-PCR showed the upregulation of osteogenic related in differentiated hPDLSCs. Scale bar: 10 μm. ** *p* < 0.01.

Human PDLSCs showed the positivity for stemness and mesenchymal surface markers, as CD29, CD73, CD90 and CD105 and the negativity for CD14 and CD34, hematopoietic antigens (Figure 1A). As reported by Dominici et al., to define the mesenchymal profile the cells must also are able to adhere on a plastic substrate and are able to differentiate into a mesengenic lineage. As reported in Figure 1B, hPDLSCs cultured on a plastic Petri dish are able to adhere on the bottom and showed a fibroblast-like morphology. The positive staining of Alizarin red S solution showed the capacity to differentiate into osteogenic lineage (Figure 1C). These data were validated by the gene expression; RUNX2 and ALP were upregulated in cells maintained under osteogenic conditions when compared to the undifferentiated cells (Figure 1D).

### 3.2. hPDLSCs Adhesion Capability on Implant Surfaces

**Figure 2:**
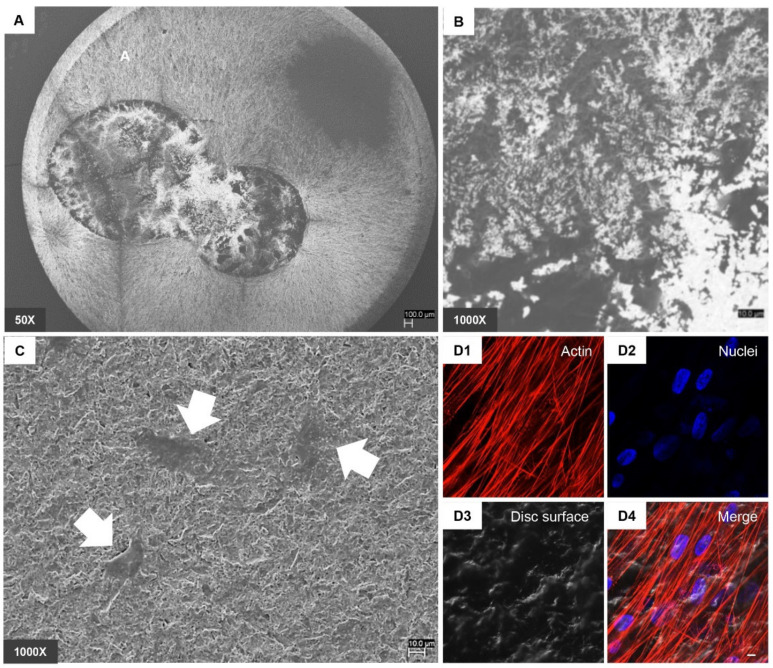
(**A**) SEM observations of CTRL surface without cells captured at 50× magnification. (**B**) SEM observations of CTRL surface without cells captured at 1000× magnification. (**C**) SEM observations of hPDLSCs cultured on CTRL surface captured at 1000× magnification. (**D1**–**D4**) CLSM observation of hPDLSCs cultured on CTRL surface. (**D1**) Cytoskeleton actin stained with Alexa Fluor 594 phalloidin and observed under red fluorescence channel; (**D2**) Nuclei stained with TOPRO and observed under blue fluorescence channel; (**D3**) CTRL surface observed under light transmission channel; (**D4**) merged image of above mentioned channels. Scale bar: 20 μm. White arrows indicate adherent cells on CTRL surface.

**Figure 3:**
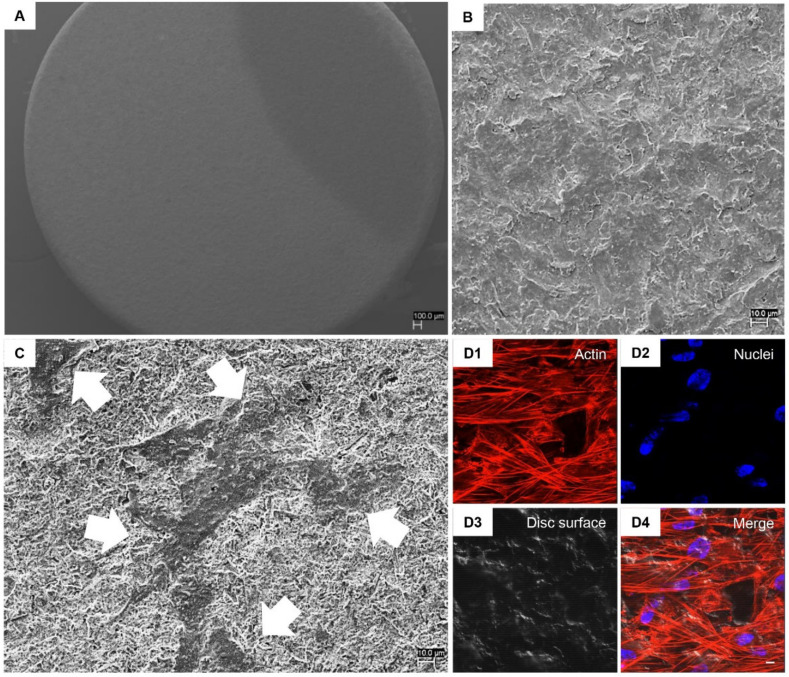
(**A**) SEM observations of TEST surface without cells captured at 50× magnification. (**B**) SEM observations of TEST surface without cells captured at 1000× magnification. (**C**) SEM observations of hPDLSCs cultured on TEST surface captured at 1000× magnification. (**D1–D4**) CLSM observation of hPDLSCs cultured on TEST surface. (**D1**) Cytoskeleton actin stained with Alexa Fluor 594 phalloidin and observed under red fluorescence channel; (**D2**) Nuclei stained with TOPRO and observed under blue fluorescence channel; (**D3**) TEST surface observed under light transmission channel; (**D4**) merged image of above mentioned channels. Scale bar: 20 μm. White arrows indicate adherent cells on TEST surface.

To demonstrate the capacity of hPDLSCs to adhere on titanium disc surface, SEM and CLSM system have been used. Figure 2 and Figure 3 in Section A and B showed the CTRL and TEST surface morphology without cells observed at different magnification, 50× and 1000× respectively. Figure 2 and Figure 3 in Section C showed the capacity of hPDLSCs to adhere on CTRL and TEST surface disc. Cells showed no evident morphological change. To better evaluate the adhesion capacity and cytoskeleton arrangement at intracellular level, the immunofluorescence acquisitions have been performed. Cells cultured on CTRL and TEST surface showed the presence of evident actin fibres and on TEST surface cells were distributed in a multilayer way.

### 3.3. Fibronectin, Laminin, N-Cadherin and RUNX2 Overexpression in TEST Surface Titanium Implant by CLSM

**Figure 4:**
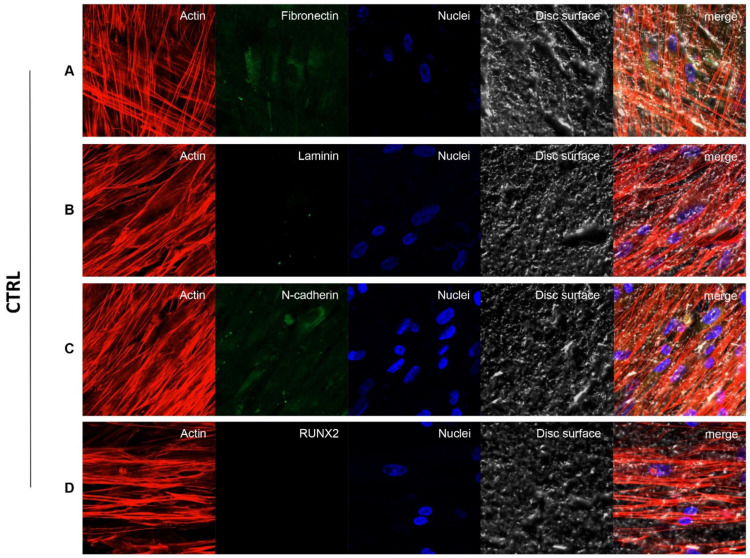
Human PDLSCs cultured on CTRL titanium implant surface were observed after 8 weeks of culture. Cytoskeleton actin was stained in red fluorescence; specific markers (**A**) Fibronectin, (**B**) Laminin, (**C**) N-cadherin and (**D**) RUNX2, were stained in green fluorescence; nuclei were stained in blue fluorescence. Scale bar: 10 µm.

**Figure 5:**
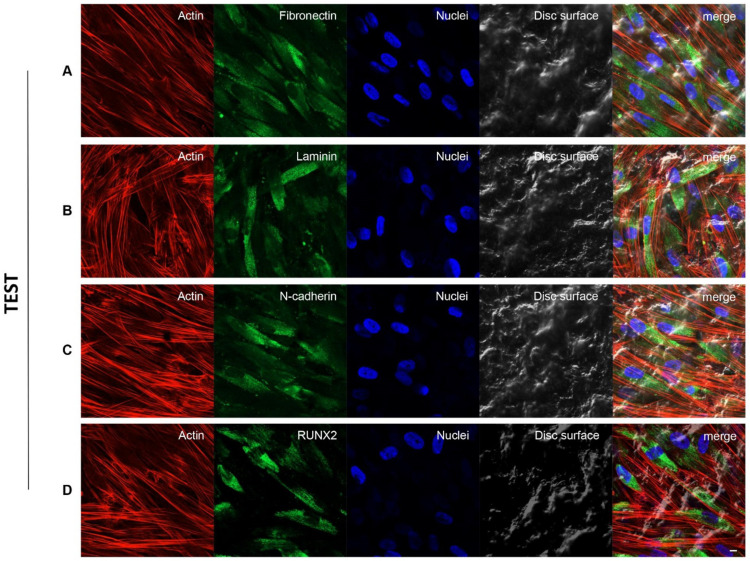
Human PDLSCs cultured on TEST titanium implant surface were observed after 8 weeks of incubation. Cytoskeleton actin was stained in red fluorescence; specific markers (**A**) Fibronectin, (**B**) Laminin, (**C**) N-cadherin and (**D**) RUNX2, were stained in green fluorescence; nuclei were stained in blue fluorescence. Scale bar: 10 µm.

Figure 4 and Figure 5 exhibited fluorescence images of the cytoskeleton actin (phalloidin, red), the specific marker (Alexa Fluor 488, green) and the nuclei (TOPRO, blue) of hPDLSCs seeded on CTRL and TEST specimens captured after 8 weeks of culture. The cells adhered and spread well with a spindle fibroblast-like shape on all samples which revealed that the different surface treatment did not affect the adhesion capability. The CLSM observation showed a higher RUNX2 expression level for hPDLSCs seeded on TEST compared to CTRL, suggesting a better capability of hPDLSCs seeded on TEST surface to differentiate versus the osteogenic lineage after 8 weeks of culture (Figure 4 and Figure 5). Furthermore, hPDLSCs cultured on TEST implant surface evidenced a higher expression of Fibronectin, Laminin and N-cadherin compared to cells seeded on CTRL sample (Figure 4 and Figure 5).

### 3.4. RUNX2, ALP and ECM Components: Gene and Protein Expression

The graph showed the gene expression of RUNX2, ALP, VIM, FN1, CDH2 and LAMB1, FAK and ITGB1 evaluated by RT-PCR after 8 weeks of culture (Figure 4). The hPDLSCs seeded on TEST reported a significant higher expression of RUNX2 in comparison with hPDLSCs seeded on CTRL disk surface. Furthermore, hPDLSCs cultured on TEST surface evidenced a significant higher gene expression of FN1 and LAMB1 compared to the CTRL surface and a slight increase in relative gene expression level of CDH2 and ITGB1 in TEST disk in comparison with CTRL surface. Conversely, no significant difference in the gene expression levels of ITGB1 in hPDLSCs on CTRL and TEST titanium disk was noticed.

**Figure 6:**
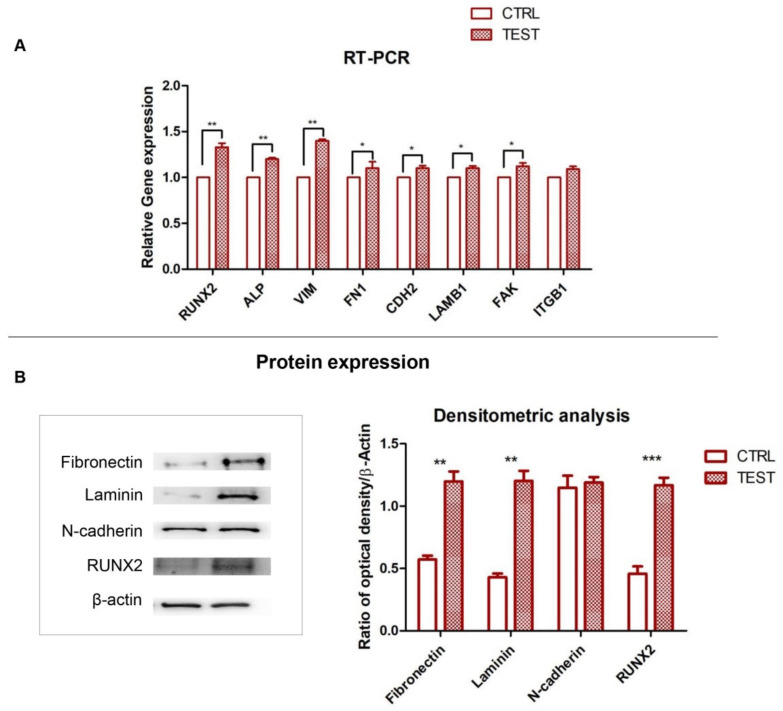
(**A**) Graph of RT-PCR showed the mRNA levels of RUNX2, ALP, VIM, FN1, CDH2, LAMB1, FAK and ITGB1 in cells cultured on CTRL and TEST surface. * *p* < 0.05; ** *p* < 0.01. (**B**) Protein level expression of Fibronectin, Laminin, N-cadherin and RUNX2 in cells cultured on CTRL and TEST surface. β-actin was used as a housekeeping protein. Graph bars represent the densitometric measurements of proteins bands expressed as integrated optical intensity with the mean of three separate experiments. The error bars in these graphs showed the standard deviation (±SD). Densitometric values analysed by ANOVA showed significant differences. ** *p* < 0.01; *** *p* < 0.001. Please refer to Full Western blot in Supplementary.

Gene expression confirmed the qualitative results obtained by CLSM observations. Protein expression of specific bands of Fibronectin, Laminin, N-cadherin and RUNX2showed an over expression in hPDLSCs cultured on TEST compared to the cells seeded on CTRL surface (Figure 6). Moreover, the densitometric analysis reported a similar trend obtained by gene expression results (Figure 6).

## 4. Discussion

In the present work, the aim was to evaluate the efficacy of different titanium surfaces treatment on hPDLSCs, used as an in vitro cell system, to assess the cell morphology, adhesion capacity and the ECM components release. In this context, the hPDLSCs were cultured on CTRL and TEST surfaces in order to investigate the cell morphological features by means of SEM and CLSM observations.

Human PDLSCs cultured on TEST surface showed a slightly better morphology compared to CTRL sample. TEST surface allowed the cells to adhere and grow onto the surface, thereby enhancing the ability of implants to bind to surrounding bone tissue. The dual acid surface treatment has been well documented to have appropriate osteoinductive ability [31,32].

A suitable implant surface should exhibit both osseoconductive and osseoinductive characteristics, encourage peri-implant bone wound healing and subsequently the development of well-structured mature bone with a high proportion of bone-to-implant interaction [20,33,34]. The biomaterials’ chemical composition and their degree of surface roughness may impact the biological responses which influence the binding of proteins, cell attachment, proliferation and differentiation and the osteoblast cell maturation [35]. In search of novel effective strategies to improve osseointegration, ECM became a very attractive point of interest. ECM exemplifies the natural surroundings of implants in bone [36]. Many researchers focus their attention on the emerging concept of ECM which is able to integrate and respond to the physical and chemical environmental changes. ECM can be considered as a scaffold for the cells, a reservoir for growth factors and cytokines, and it modulates the cell activation status and turnover [22,37].

In the current study we analysed the release of ECM components critical for osseointegration and osteogenesis. Gene expression level of ECM components such as VIM, FN1, CDH2, LAMB1, FAK and ITGB1 was assessed, in addition to the osteogenic related markers RUNX2 and ALP. The RT-PCR data showed an increased expression of the above-mentioned genes in hPDLSCs grown on the TEST implant surface, which indicated that the roughness surface was able to induce the release of ECM molecules. ECM components constitute a dynamic and complex network structure with specific physiological and biochemical properties. ECM proteins regulate the adhesion, migration, proliferation and osteogenic differentiation of stem cells during bone regeneration [38,39].

The interactions between cells and ECM are mediated by integrins, which serve as signal transducers for regulating cell activities. Integrins, a superfamily of cell adhesion receptors that bind to ECM ligands, cell-surface ligands and soluble ligands are able to interact with the cytoskeleton actin to anchor the cells and to function as signal carriers, which give cues to the cell about the ECM state of spatial cell biology [40,41]. The association of integrins with the cellular signalling network initiates downstream signalling cascades such as FAK pathway [42]. Focal adhesion complexes are key structures participating in the interactions between cells and surfaces of biomaterials and may affect cell morphology, proliferation and differentiation [43].

The β1 sub-family integrins are the major expressed integrins in osteoprogenitors and osteoblasts. In particular, the fibronectin receptor integrin α5β1 is known to play a key role in the osteogenesis of MSCs and it is a common mediator to promote cell attachment and crucial for tissue repair [44,45]. The development of a strategy that modifies the dental implant surface in order to modulate the interaction between integrins and the ECM represents an important tool for the therapies related to bone tissue in dentistry and medicine.

Lopes et al. demonstrated that the surface topographic characteristics modulated the FAK expression, a relevant marker for osteoblastic differentiation of hPDLSCs grown on titanium surfaces; meanwhile, FAK inhibition down-regulated the gene expression of key bone markers and ALP activity in cells grown on different evaluated surfaces [46].

Recent studies have reported that Vimentin also showed a key role in the cellular adhesion process that regulates the integrin functions. Vimentin is one of the major intermediate filaments (IFs) protein in mesenchymal cells; indeed, IFs are fundamental in the adhesion and in the cell–cell interactions through their association with hemidesmosomes and desmosomes [47]. The purpose of the hPDLSCs culture on titanium implant surface represents an innovative approach to study the ECM molecules release. Human PDLSCs are MSCs residing in the periodontal tissues and are able to contribute to the periodontal tissue regeneration [48]. In fact, hPDLSCs showed the features of the MSCs, such as self-renewal, immunomodulatory and multi-tissue differentiation capacity. They showed the positivity for mesenchymal surface markers (as CD 29, CD73, CD90 and CD105) and are able to differentiate toward the osteogenic lineage [49]. The results obtained by immunofluorescence and biochemical analyses suggest that the TEST surface may promote the secretion of ECM components fibronectin and laminin which may induce hPDLSCs osteogenesis and implant osseointegration as evidenced from the higher expression of RUNX2 in TEST with respect to CTRL sample. Furthermore, the slight increase in the expression level of N-cadherin could contribute to the enhancement of hPDLSCs osteogenesis and thus lead to more bone matrix deposition on the titanium implant.

ECM proteins, such as laminin, fibronectin and N-cadherin were found to be increased greatly in hPDSLCs cultured on TEST. Fibronectin belongs to one of the principal adhesion protein family for eukaryotic cells, which plays a key role in binding to integrins as well as other extracellular components including collagen, fibrin and proteoglycans [50]. N-cadherin is a key factor in directing cell–cell interaction during the mesenchymal condensation and it is also considered as a mimetic peptide that enhanced the osteogenic differentiation of the MSCs, leading to bone matrix deposition on the titanium implants [51]. Laminins are glycoproteins and major structural components in the basal lamina and showed a critical role to cell adhesion, differentiation and migration [52].

Despite the limitations of the present in vitro study, relevant and positive outcomes have been obtained. Taken together, these findings highlighted that initial cell adhesion and spreading play an important role in early stage of wound healing to lead the bone formation and the osseointegration. Understanding the mechanisms of the release of ECM components and its regulation is essential for developing novel strategies in the field of tissue engineering and regenerative medicine. The future of dental implantology aims at developing an implant surface with specific topography. The approach proposed in the present study provides an understanding of the interactions between proteins, cells and implant surface. The local release of ECM proteins could stimulate the bone formation process. This strategy may ultimately enhance in vivo the osseointegration process of dental implants for their immediate loading and long-term success.

## 5. Conclusions

Our results demonstrated the relevance of ECM components’ interactions between oral MSCs and the titanium implant surface topography. The application of the treated dental implant surfaces could improve the biological performance of titanium implants that significantly enhance the release of ECM components; this represents the necessary step in the early stage of osseointegration. The development of titanium surfaces that are able to regulate the ECM components release could positively impact the process of implant osseointegration.

## Data Availability

Data are available upon request.

## Supplementary Materials

The following are available online at www.mdpi.com/article/10.3390/biology10090931/s1.

## Abbreviations

ECM	extracellular matrix
hPDLSCs	human periodontal ligament stem cells
ITGB1	integrin beta-1
FAK	focal adhesion kinase
FN	fibronectin
CDH2	N-cadherin
LAMB1,	laminin
ALP	alkaline phosphatase
RUNX2	runt related transcription factor 2
SEM	scanning electron microscopy
CLSM	confocal laser scanning microscopy
VEGF	vascular endothelial growth factor
PBS	phosphate buffered saline
RT-PCR	real time–polymerase chain reaction
MSCs	mesenchymal stem cells
RT	room temperature
ECL	chemiluminescence detection method
IFs	intermediate filaments
VIM	vimentin
