# Correction: Marconi et al. Human Periodontal Ligament Stem Cells Response to Titanium Implant Surface: Extracellular Matrix Deposition. *Biology* 2021, *10*, 931

**DOI:** 10.3390/biology12020306

**Published:** 2023-02-14

**Authors:** Guya Diletta Marconi, Luigia Fonticoli, Ylenia Della Rocca, Thangavelu Soundara Rajan, Adriano Piattelli, Oriana Trubiani, Jacopo Pizzicannella, Francesca Diomede

**Affiliations:** 1 Department of Medical, Oral and Biotechnological Sciences, University “G. d’Annunzio” Chieti-Pescara, 66100 Chieti, Italy; 2 Department of Innovative Technologies in Medicine & Dentistry, University “G. d’Annunzio” Chieti-Pescara, 66100 Chieti, Italy; 3 Department of Biotechnology, Karpagam Academy of Higher Education, Coimbatore 641021, India; 4 “Ss. Annunziata” Hospital, ASL 02 Lanciano-Vasto-Chieti, 66100 Chieti, Italy

## Figure/Table Legend

In the original publication [1], there was a mistake in the legend for “Figures 3C, 4D and 5A”. The figures were already published in our paper in *Frontiers and Cell Development*; due to our inattention, the citation [14] was not reported in the paper in *Biology* and should be added.

The authors state that the scientific conclusions are unaffected. This correction was approved by the Academic Editor. The original publication has also been updated.
