# Supplementary figures and images for: Human Periodontal Ligament Stem Cells Response to Titanium Implant Surface: Extracellular Matrix Deposition
Source: Biology (Basel). 2021 Sep 18;10(9):931. doi: 10.3390/biology10090931 (PMC8470763; doi:10.3390/biology10090931)

Fibronectin

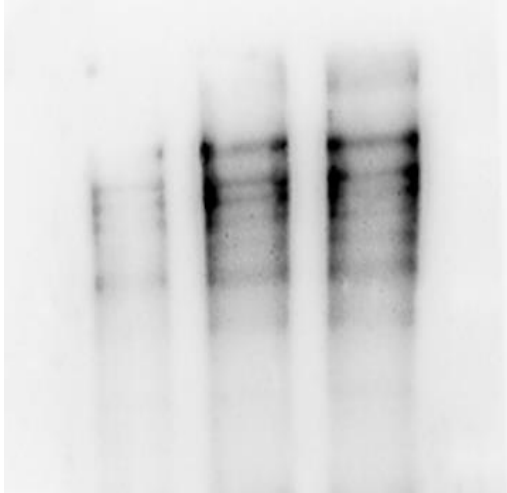

RUNX2

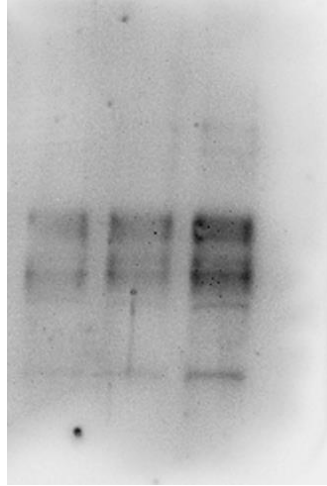

Beta actin

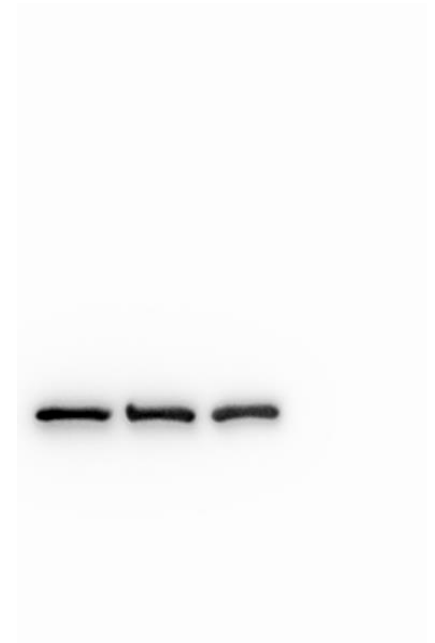

N cadherin

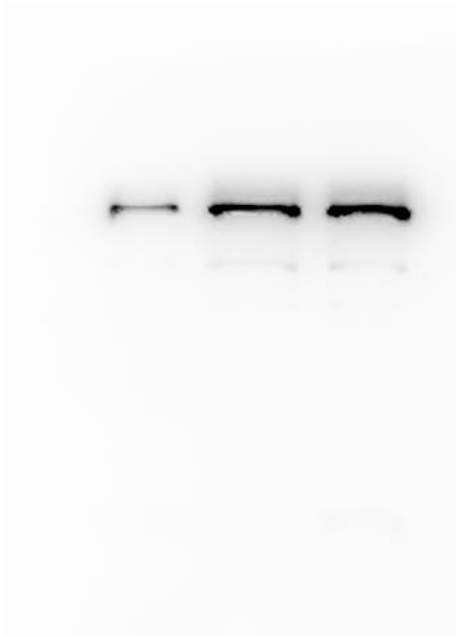

Laminin

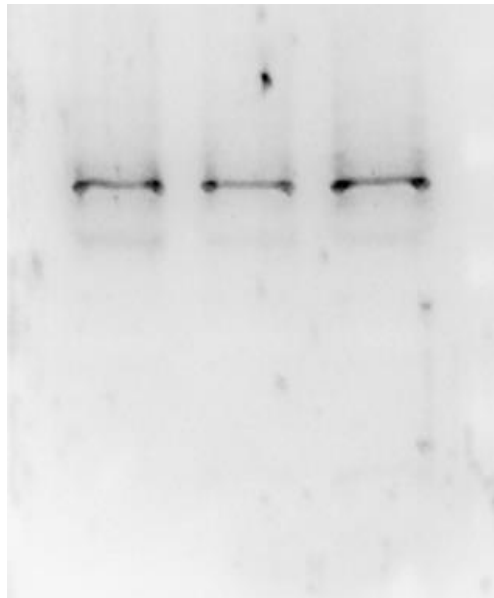

Supplement: Supplementary file 1 [file biology-10-00931-s001.zip › biology-1340221-supplementary.pdf]
